# Fragile DNA Repair Mechanism Reduces Ageing in Multicellular Model

**DOI:** 10.1371/journal.pone.0036018

**Published:** 2012-05-02

**Authors:** Kristian Moss Bendtsen, Jeppe Juul, Ala Trusina

**Affiliations:** Niels Bohr Institute/CMOL, University of Copenhagen, Copenhagen, Denmark; University of Maribor, Slovenia

## Abstract

DNA damages, as well as mutations, increase with age. It is believed that these result from increased genotoxic stress and decreased capacity for DNA repair. The two causes are not independent, DNA damage can, for example, through mutations, compromise the capacity for DNA repair, which in turn increases the amount of unrepaired DNA damage. Despite this vicious circle, we ask, can cells maintain a high DNA repair capacity for some time or is repair capacity bound to continuously decline with age? We here present a simple mathematical model for ageing in multicellular systems where cells subjected to DNA damage can undergo full repair, go apoptotic, or accumulate mutations thus reducing DNA repair capacity. Our model predicts that at the tissue level repair rate does not continuously decline with age, but instead has a characteristic extended period of high and non-declining DNA repair capacity, followed by a rapid decline. Furthermore, the time of high functionality increases, and consequently slows down the ageing process, if the DNA repair mechanism itself is vulnerable to DNA damages. Although counterintuitive at first glance, a fragile repair mechanism allows for a faster removal of compromised cells, thus freeing the space for healthy peers. This finding might be a first step toward understanding why a mutation in single DNA repair protein (e.g. Wrn or Blm) is not buffered by other repair proteins and therefore, leads to severe ageing disorders.

## Introduction

In humans, ageing is associated with the gradual deterioration of physiological functions, – blood vessels become less flexible, bones turn brittle, muscle mass is lost, and the immune system becomes more vulnerable to infections.

At the cellular level, ageing is thought to be caused, at least in part, by the accumulation of unrepaired damage to mitochondrial or nuclear DNA [1]–[3]. Damage can be induced by intrinsic factors, such as reactive oxygen species, or have an external cause, such as exposure to UV-light or toxic chemicals [4].

At the organism level the relevant time scale for ageing is that of a human life. In slowly proliferating cells, such as neurons or bone cells with lifespans well over 30 years, ageing can be contributed to the accumulation of damage in individual cells. For highly proliferating cells with typical lifespans of a few days [5], unrepaired DNA damage causes cell cycle arrest and consequently apoptosis. Erroneously repaired DNA damage can result in a mutation and in highly proliferating cells the mutation is passed down through the lineage until the cell line reaches the Hayflick limit. The Hayflick limit can be estimated to around 50 cell divisions, corresponding to a few years [6], [7]. After this, all information of the mutation is lost.

In fast turnover tissue, ageing can therefore not be a direct consequence of DNA damage accumulated in individual cells or mutations transmitted through the lineage of cells. As the capacity for regeneration and renewal of tissue is dependent on the population of somatic stem cells, which have much longer lifespans [8], [9], ageing in highly proliferating cells can be explained by the progressive decline of stem cell function. The decline of stem cell function in responds cell homeostasis has previously been mathematically modelled by Wodarz [10].

Somatic stem cells are kept in a low-activity quiescent state to minimize the use of ATP and thereby reduce the production of reactive oxidative species. When cell renewal is needed to maintain homeostasis, stem cells move from the quiescent state into a proliferating state, where the risk of acquiring DNA damage increases [11], [12]. Hence, the longer proliferating cells can maintain a level of functionality comparable to that of a young organism without requiring renewal from the stem cell pool, the slower the decline of the stem cell pool will be, which, in turn, will slow down ageing of the organism.

One of the cell functions that has been observed to have a great impact on ageing is the ability to repair DNA damage. Several diseases that compromise this ability are associated with mutations in DNA repair proteins and show symptoms of premature ageing. Such diseases include Werner syndrome, ataxia telangiectasia, and Bloom syndrome [13], [14]. Interestingly, it has also been shown in several organisms that the capacity of DNA repair declines with age [15] and that the mutation frequency increases with age [16].

In this work we focus on DNA damage leading to mutations that impair the ability to repair future genotoxic damage. We introduce a simple model to investigate how a population of highly proliferating cells exposed to genotoxic damage may maintain a high function without renewal from the stem cell pool.

**Figure 1:**
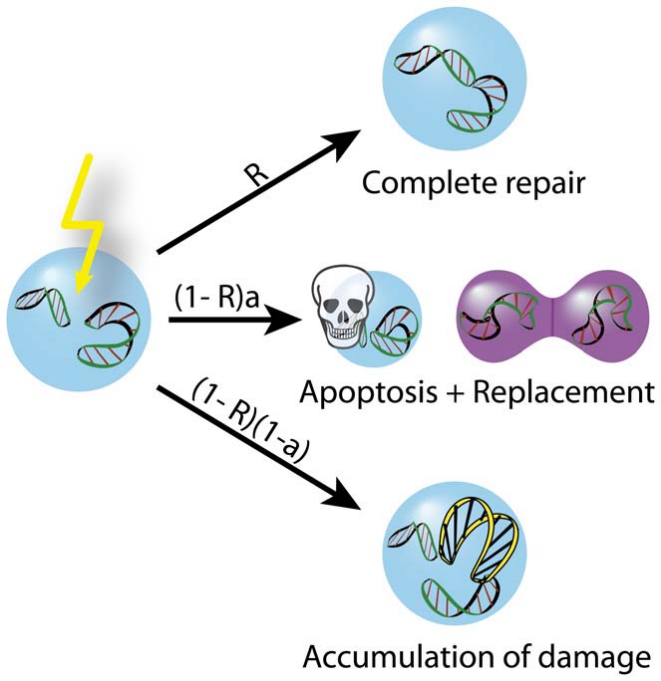
When a cell acquires DNA damage, it may respond in one of three ways: i) Complete repair of the damage with probability *R*, ii) Apoptosis with probability 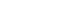 **in which case another cell divides to keep the number of cells constant, iii) Accumulation of the damage through a mutation, reducing the rate of repair to** 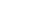

In our model, cells continually acquire DNA damage. The result of the damage is modeled by three possible outcomes: a) repair, b) apoptosis and c) mutation. For simplicity we choose not to model the senescence or the influx of new cells from the stem cell pool explicitly. However, these are considered implicitly by monitoring the number of cell replications and the total number of cells at any point in time. Regarding the three outcomes a few remarks are in order: a) The rate of repair, *R*, is specific to each cell, but all cells are initiated with the same rate 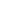. b) If the cell is unable to fully repair the damage, it has a probability *a* of going apoptotic. In case of apoptosis, another cell divides to keep the total number of cells constant and the daughter cells inherit the repair rate of the parent cell. c) If the damage is not fully repaired and the cell does not go apoptotic a mutation occurs impairing the ability of the cell to repair future genotoxic damage by reducing the repair rate to 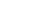. The parameter Δ can be interpreted as the fragility of the DNA repair mechanism. If Δ is large, cells with unrepaired DNA damage will have a greatly reduced ability to repair future genotoxic damage (see figure 1 and *Methods*).

## Results

**Figure 2:**
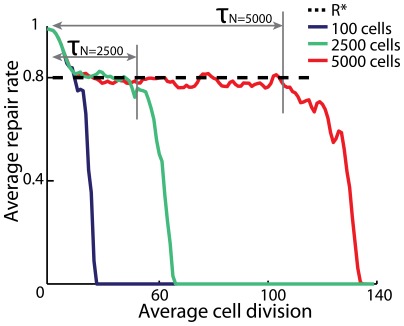
The average repair rate of the system drops as cells irreversibly accumulate mutations. Since cells with a low rate of repair are more likely to go apoptotic, a temporary steady state exists where the repair rate of the system fluctuates around an average value of 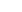 The length of time, 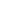 before the system leaves this temporary state increases drastically with the size *N* of the system. If 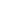 is large, a high rate of repair may be maintained without renewal from the stem cell pool, and consequently the organism ages more slowly. The simulation is carried out with parameters 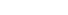 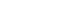 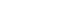 The median of 20 simulation runs is showed.

The average repair rate of the system, 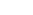 starts out at the maximum value 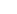 and then decreases as cells accumulate mutations. Since mutations are irreversible, the repair rate of each cell will inevitably drop to zero. However, a temporary steady state exists where the repair rate of the system fluctuates around an average value of 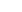 In this state, the average repair rate is maintained because cells with a low repair rate are more likely to go apoptotic and be replaced by cells with a higher repair rate (see figure 2).

The system will leave the temporary steady state after a time 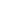 at which point the average rate of repair drops drastically. Thus, 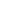 can be interpreted as the time after which introduction of new cells from the stem cell pool is needed to sustain a high rate of repair. Since the stem cell pool accumulates less damage (and consequently mutations) when the rate of proliferation for stem cells is reduced, a large 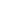 corresponds to a slower ageing of the organism [17], [18].

**Figure 3:**
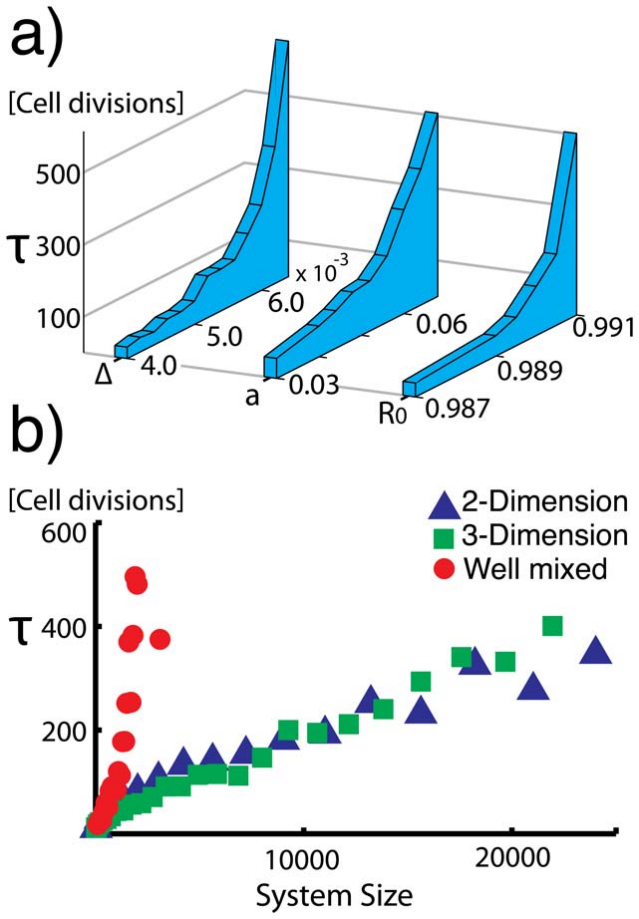
a) The time 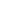 spent in steady state increases with the initial repair rate 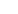, rate of apoptosis *a*, and fragility of the repair mechanism Δ. (Parameters that are not varied are set to the values given in the caption of figure 2) b) For the well mixed system, the time spent at steady state, 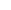 increases drastically with system size *N*. For spatially structured systems the increase is linear. (Parameters used: 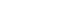 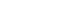 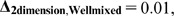 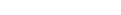), 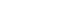

When the initial rate of repair 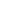 is high, both the steady state rate of repair 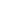 and the time spent in this state 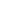 increases (see figure 3). Since new, undamaged tissue is produced by somatic stem cells, the initial rate of repair can be thought of as the repair rate of these cells. Thus, the repair rate of stem cells is not only important for avoiding DNA damage in the stem cell pool, but also for retaining a high level of function in tissue cells.

This result is interesting from the perspective of tissue specific DNA repair capacity. There are several studies showing that the repair capacity in testis nuclei and mitochondria are higher than in any other tissue. This could be explained by the high expression level, in the testes, of many DNA repair genes. [1], [19], [20]. Our results predict that the higher repair capacity of this tissue would minimize the load on the stem cell pool.

A high rate of apoptotis, *a*, decreases the risk of accumulating damage and, therefore, also increases both 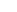 and 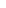 (see figure 3). Surprisingly, increasing the rate of apoptisis does not cause more cells to go apoptotic in the system. The increase in the average rate of repair in the steady state precisely balances out the increased risk of apoptosis when the repair fails. Hence, a higher apoptosis rate does not increase the rate of cell divisions needed to maintain homeostasis. Consequently, the Hayflick limit in the tissue is not reached faster for a higher apoptosis rate.

When the fragility of the repair mechanism, Δ, is high, cells with unrepaired DNA damage will be more likely to go apoptotic when exposed to further DNA damage. This allows the system to maintain a high rate of repair longer, the more fragile the DNA repair mechanism is to DNA damages (see figure 3).

In a well mixed system, the time 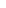 increases fast with the number of cells *N*. In a 2 or 3 dimensional system, where apoptotic cells can only be replaced by neighbouring cells dividing, the time 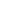 increases linearly with increasing *N* (see figure 3b). Hence, for cells that are spatially structured such as skin or organ tissue, renewal from the stem cell pool per cell is constant regardless of system size. For a well mixed system, such as the circulatory system, our model suggests a cooperativity between dividing cells (e.g. lymphocytes) that allows large systems to sustain a high rate of repair without increased renewal from the stem cell pool.

### Theoretical Analysis

In this section, we investigate the model analytically in order to find the configuration of the temporary steady state for the well mixed system where all cells are neighbours.

The state of the system can be characterized by the number of times *i* each cell has accumulated DNA damage. We therefore consider a number of cells 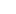 with a repair rate of 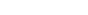 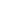 will decrease when the cells fail to repair after being exposed to DNA damage. Conversely, 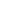 will increase when these cells replicate after another cell has gone apoptotic, which will happen at rate 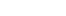 Cells with 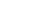 mutations will, in addition, increase in numbers when cells of repair rate 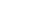 mutate one more time. These considerations lead to the dynamical equations of the system.
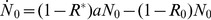
(1)

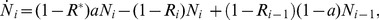
(2)where the dot represents a time derivative and 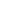 is the average repair rate of the temporary steady state. Demanding that the time derivatives vanish turns (1) into an expression for the average repair rate, which can be inserted into (2) to give a recurrence relation for the temporary steady state.
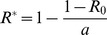
(3)

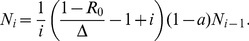
(4)This recurrence relation can be solved to yield a general expression for the number of cells having accumulated DNA damage *i* times.
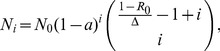
(5)where the last factor is a binomial coefficient and 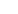 is both a normalization factor and the number of undamaged cells, which is seen by setting 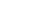 The expression (5) can be verified by insertion into (4) using the identity for binomial factors 
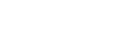
.

**Figure 4:**
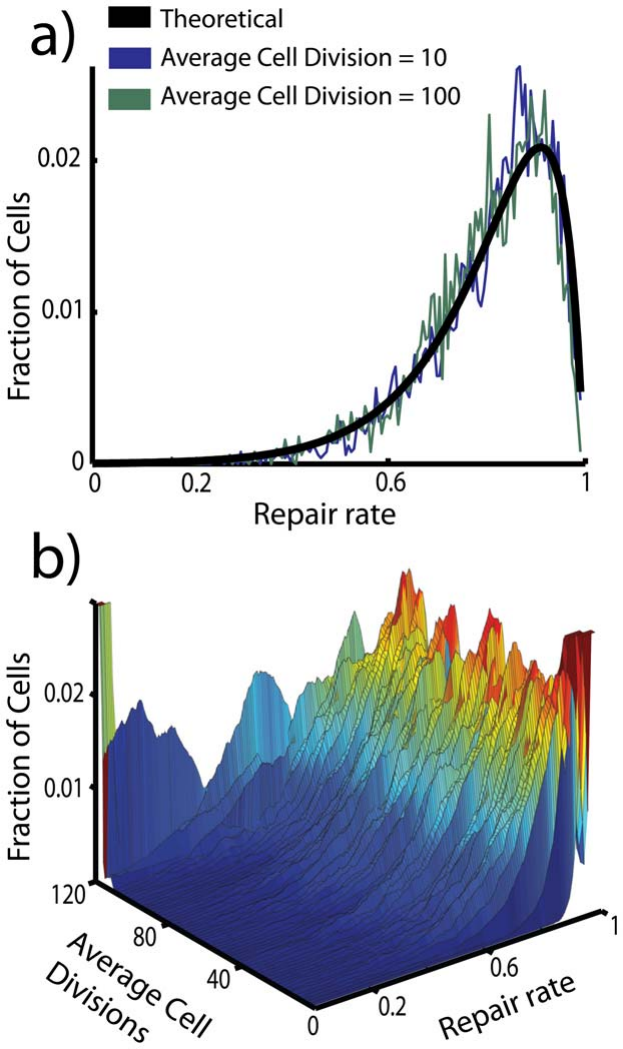
a) The theoretically predicted distributions of repair rates in the well mixed systems, given by (5), is seen to agree well with the actual distribution during the temporary steady state. **b)** The distribution of repair rates as a function of time. (All parameters are set to the values given in the caption of figure 2.)

Summing all 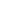 should give the total number of cells in the system *N*, which leads us to an expression for the normalization factor
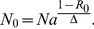
(6)Notice that we here have summed over all *i* and not truncated the sum at 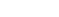 after which the repair rate becomes negative. However, for small Δ, states with 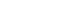 contain virtually no cells (see figure 4a).

We can confirm that a steady state exists by showing that the average repair rate of the system 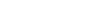 is in fact given by (3). Figure 4 shows the steady state distribution (5), as well as the development of the distribution over time, for 5000 cells and with the same set of parameters as the simulation in figure 2. The steady state mean repair rate (3) is in perfect agreement with simulations for the well mixed system. In spatially structured systems 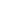 takes a lower value.

We are interested in finding the average lifetime 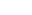 of cells in the well mixed system. That is, the number of time steps the average cell has gone through at the time it goes apoptotic. At each time step an average cell has the probability 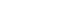 of going apoptotic. Thus, the average cells will be alive for
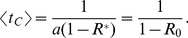
(7)Notice that the average lifetime is depends on the initial repair rate 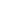, meaning that increasing the rate of apoptosis *a* does not increase the number of cells going apoptotic in the system, which agrees with simulations.

It can be shown that the steady state is attractive: Introducing the pertubation 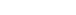 in (2) leads to 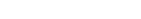, which will tend to reduce the pertubation. However, due to the finite size of the system, the number of cells that have accumulated damage *i* times will fluctuate stochastically around the distribution (5). For undamaged cells, the temporary steady state is marginally stable to first order. If the number of undamaged cells randomly fluctuates down to zero, new undamaged cells can never be reintroduced, since this would require renewal from the stem cell pool. Thus, the time 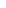 the system spends in the temporary steady state can be understood as the time passing before random fluctuations cause the number of undamaged cells to go to zero. After this, a new steady state with a lower maximum repair rate can be found, corresponding to the substitution 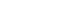 in (5). But from figure 2a we see that this steady state will be even more unstable than the previous. Therefore, the system quickly evolves to a state where all cells have accumulated the maximum amount of DNA damage in accordance with figure 2. If the temporary steady state contains a high number of undamaged cells, the relative fluctuations of these will be small, such that it becomes less likely that this number fluctuates down to zero. That is, if 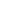 is high, the time spent in the steady state 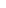 increases drastically. From (6) we see that 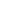 increases with both 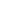 *a*, Δ, and *N*, which explains figure (3 a).

## Discussion

Ageing in fast turnover tissue of a multicellular organism can be seen as the progressive deterioration of cell function caused by wear and tear on somatic stem cells. Therefore, tissue that can maintain its function for a long time without renewal from stem cells will have a slower ageing process. The capability to repair DNA damage, preventing the formation of mutations that can be passed down through the lineage of cells, is strongly linked to both ageing and cancer [4]. We have introduced a simple model to investigate how mutations that compromise DNA repair capacity in single cells affect a whole tissue.

The model in its present form captures two important experimental observations, namely the decreased repair rate and increased mutation frequency with age [15]. It further suggests that simply by imposing the condition for tissue homeostasis – that every dying cell is replaced by a dividing neighbour cell – mutations that impair the repair mechanism are rapidly removed from the cell population such that a high repair capability is maintained at the tissue level.

There are two interesting and non-intuitive predictions resulting form our model. First, the model predicts that an initial rapid decline in repair capacity should be followed by a rather long period of persistently high repair capacity R*. In this temporary steady state, the average number of mutations to genes coding for DNA repair should remain approximately constant. Remarkably the experimental studies on mutation frequencies in mice by Busuttil et al., [16] seem to suggest similar temporal behavior: the initial rapid increase in mutation frequencies (young mice) is followed by constant or nearly constant mutation frequencies (older mice).

Second, this time-span of this high repair capacity is higher when repair mechanism is fragile. Thus, the ageing of the organism will slow down, if mutation inflicted by DNA damage greatly decreases the capability to repair future DNA damage. Such a fragile DNA repair machinery would allow for quick removal of cells with compromised DNA repair, leaving space for healthier cells to fill in the gaps. This result is closely related to the recent observations by Baker et al [21], where removal of senescent cells was shown to delay tissue dysfunction and slow down ageing.

The time that the high repair capacity at the tissue level can be self-sustained for increases drastically with system size in a well mixed system. Furthermore, the time increases with the initial capability to repair DNA damage, the rate of apoptosis, and the fragility of the repair mechanism. Thus, if mutations inflicted by DNA damage greatly decreases the capability to repair future DNA damage, this will slow down ageing of the organism. Such a fragile DNA repair machinery would allow for quick removal of cells with compromised DNA repair, leaving space for healthier cells to fill in the gaps. This result is closely related to the recent observations by Baker et al [21], where removal of senescent cells was shown to delay tissue dysfunction and slow down ageing.

Our model further predicts that: The size, initial capability to repair DNA damage, the rate of apoptosis, are all increasing the average time the repair capability can be sustained for. This time also depends on the tissue configuration – the closer the tissue is to the conditions of a well mixed system, the longer the duration of high repair capacity is.This implies that cells may “coorperate” in keeping a high function, and that a 3-dimensional tissue, such as organs, may need a smaller influx of stem cells than 2-dimensional tissue, such as skin.

We find that the average lifetime of cells in the model is independent of the rate at which damaged cells go apoptotic. It should be noted that, since our model only takes into account damages to the parts of the DNA involved in the DNA repair mechanism, the model is not able to distinguish a successful repair from damage to the genome that does not, directly or indirectly, influence DNA repair pathways. If the rate of apoptosis is increased when damages occur in other parts of the DNA, the average lifetime of cells will be decreased. It is still an open question to what extent apoptosis affects the process of normal ageing [22].

The number of spontaneous DNA damages inflicted in every cell every day may be as high as 100,000 [23]. However, DNA damages can also occur during the replication process, leading to additional mutations not considered in this model. Apart from accumulation of DNA damages, tissue deterioration is associated with an increase in the amount of senescent cells in the organism. That is, the number of cells which, after reaching their Hayflick limit, go into permanent cell cycle arrest [17]. Also, the mechanisms of ageing and the defence mechanisms against cancer are highly interconnected [4]. Incorporating replication damages, senescence, and cancer into the model may provide new knowledge of the development of ageing and the connection between ageing and cancer.

## Methods

In each time step of the model, a random cell out of *N* acquires a DNA damage. The cell can react in three ways: (i) The damage can be fully repaired with probability *R*. The repair rate *R* is specific to the individual cell. (ii) If the cell is unable to repair the damage, it can go apoptotic with probability *a*. In this case another cell divides to replace it, keeping the total number of cells constant. (iii) Else, the cell accumulates the damage through a mutation in the DNA, reducing its ability to repair future genotoxic damage from *R* to 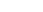


When a cell divides to take the place of a cell that has gone apoptotic, the daughter cell inherits the repair rate from the parent cell. When investigating 2 or 3 dimensional systems, the cells are located on a square lattice and apoptotic cells can only be replaced by neighbouring cells dividing.

All cells are initiated with the same repair rate 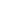. The parameter Δ can be interpreted as the fragility of the DNA repair mechanism. If Δ is large, cells with unrepaired DNA damage will have a greatly reduced ability to repair future genotoxic damage.
